# Exploring Children's Perceptions of their Local Environment in Relation to Time Spent Outside

**DOI:** 10.1111/chso.12217

**Published:** 2017-03-19

**Authors:** Felicity Hayball, Paul McCrorie, Alison Kirk, Ann‐Marie Gibson, Anne Ellaway

**Affiliations:** ^1^ MRC Social and Public Health Sciences Unit University of Glasgow Glasgow UK; ^2^ University of Strathclyde Glasgow UK

**Keywords:** affordances, children, neighbourhoods, outdoors, participatory research, perceptions, physical activity

## Abstract

This study aims to understand how children perceive their environment, exploring the affordances children perceive to influence their physical activity (PA) behaviour when outside. Participants included boys and girls aged 10–12 years (n = 15) living in Scotland. Children's visual and verbal representations of their perceived environment were analysed to assess environmental determinants of PA. The findings suggested that physical affordances that offer a sense of risk were important to children's play spaces. Social affordances influenced where the children went in their environment and the features they utilised as part of play behaviour; strangers were considered threatening depending on whether the activity was recognised.

## Introduction

It is important to clarify what constitutes as physical activity (PA). PA is generally defined as any bodily movement produced by skeletal muscle that requires energy expenditure (Caspersen and others, 1985). Time spent playing outside is therefore considered PA. Evidence suggests that less than 20 per cent of children in Scotland are not achieving the sufficient levels of PA for health (Reilly and others, 2014). Children who reach the recommended PA levels are less likely to suffer from acute health problems such as low cardiovascular fitness (Dunn and others, 1999), likelihood of being overweight (Ortega and others, 2013) and high levels of adiposity (Strong and others, 2005). Children who do not reach the recommended PA levels are also more likely to suffer from chronic health problems such as diabetes (Liese and others, 2013), cardiovascular health risks, high blood pressure (Farpour‐Lambert and others, 2009) and obesity (Pedersen and Saltin, 2006). Frequent PA has also been shown to help improve psychological well‐being as it can reduce stress, anxiety and depression (Dunn and others, 2001).

Past literature has often concentrated on PA interventions during school breaks (otherwise known as recess) (Parrish and others, 2016), active travel to school (McMinn and others, 2012) and school PE classes (Hollis and others, 2016). However, research has found that sedentary behaviour increases and PA levels decrease during the afterschool period (Wickel and Belton, 2016), suggesting this is the time period that needs more attention within the research.

Studies have found that time spent outside is associated with higher PA levels (compared to staying indoors) (Cleland and others, 2008; Mackett and Paskins, 2008; Payne and others, 2013). Additionally, research has found that active play and outdoor play has potential to lead to increased habitual PA levels and moderate‐to‐vigorous PA levels in children (Gray and others, 2015). Thus, one way of increasing children's PA levels after school is through time spent outdoors. However, children will be less likely to spend time outdoors if the environment in unappealing to them. Moreover, children will not participate in outdoor play if their environment does not offer affordances that can be used for play behaviour. Establishing which factors contribute to an appealing activity‐promoting environment may help to increase children's willingness to spend time outside, and potentially their PA levels.

### Affordances

Much of the qualitative research within childhood PA literature has focused on the objective environment; asking children to talk about what is objectively within their environment. The current study has asked children to go out and document what is in their environment that influences their time outside — focusing on what children perceive in their environment. Although there are many theoretical frameworks that are often associated with PA, Gibson's theory of affordances (Gibson, 1977) is one of the few theories where the focus is on the perceptive environment. Gibson's theory of affordances suggests an interconnection between the environment and the observer (an individual). The theory contends that in order for activities to be possible, the individual must perceive them as such. For example, a piece of play equipment will only be used by a child if that child perceives it to be a usable affordance that is designed for play. Affordances that are perceived by a child may not be similar to an adolescent or an adult, hence this study aimed to clarify what types of affordances attract children to outdoor locations.

## Methods

### Participants

The participants were 15 children (5 boys and 10 girls,) from Glasgow, UK. The participants were from areas of low‐mid levels of area deprivation and lived in both rural and urban settings. As this was a pilot study, the children were sampled opportunistically, including recruiting children of colleagues, friends of those children and children from a local girl‐guiding group. The inclusion criteria were that the children had to be between the ages of 10 and 12 years old. The participants were given pseudonyms for the study to ensure anonymity. Data collection took place between October 2014 and December 2014. Ethics approval was granted by the institutions ethics committee. Each child received a ‘goody bag’ for taking part. The goody bag included health‐related objects such as a pedometer and a reflective cycle badge and was valued at under £10 each.

### Procedure

The study implemented a multi‐methodological approach, comprising photo elicitation, drawings and focus groups. These methods were further complemented by participant analysis, which will be discussed at a later stage.

### Visual environment

The visual aspect of the study was a combination of photo elicitation and drawings. The first phase comprised of a short introductory meeting with the children to discuss the study and what would be required of them, after which parent consent and child assent was obtained. Disposable cameras and sketchbooks were given to the children and they were asked to go out over the next seven days and take photographs or draw pictures of aspects of their environment that they felt were important to them and that affected the likelihood of them going outside. The children were informed that the study was primarily interested in what the children themselves perceived in the public outdoor environment. For clarification, the children were told to document aspects of the environment that were accessible to anyone, for example the park or streets.

The cameras and sketchbooks were collected one week after they were handed out. The photographs were developed and copies were taken of both the sketchbook entries and the photographs and all visual data were stored in a secure location.

### Focus groups

**Figure 1:**
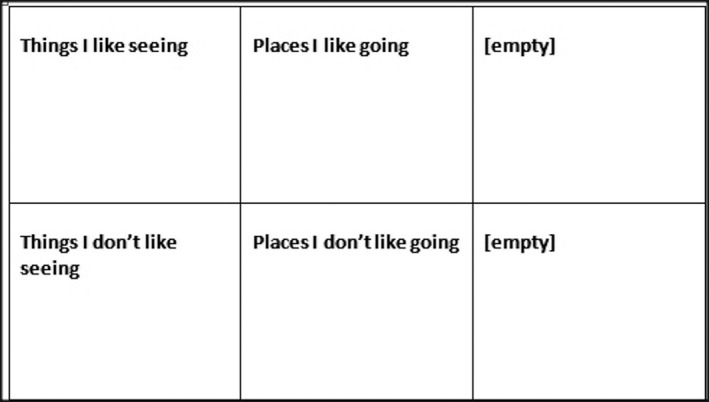
Participant analysis grid.

Before the focus group discussion, the children completed the ‘participant analysis’ task. The participant's analysis procedure was designed by the lead author. The children were asked to place their visual data into themes. Six themes were set within a large grid (Figure 1). The four predetermined themes were ‘places I like going’, ‘places I don't like going’, ‘things I like seeing’, and ‘things I don't like seeing’. Two themes were left blank for the children to self‐label inductively should they feel that the pre‐labelled themes did not reflect some, or all, of their pictures. As previously mentioned, the ‘inductive’ boxes were left in case the children did not feel their data could be placed into the pre‐labelled boxes. The children would (if applicable) place their data into the empty boxes and give the box/es a label that they felt had emerged from the data placed in the box. The purpose of the participant analysis was to allow the children to code their own work to help reduce researcher bias.

The focus group discussion commenced after the participant analysis had been completed. The format of the focus group was a discussion regarding the pictures the children had placed in each box, the subject matter of the picture, what the picture represented, why it had been placed in that specific box, and comparisons between the children's choices and settings. The first focus group comprised of six girls and lasted 45 minutes. The second focus group comprised of four boys and lasted 1 hour and 30 minutes. The third focus group comprised of one boy and four girls and lasted 2 hours. We decided to implement both same‐sex and mixed sex focus groups in order to explore whether it might influence how open and/or conversational the children were as this could potentially impact the findings and might be important to understand for future studies. After all three focus groups were completed, it did not appear that children of either gender were influenced by the gender of the other focus group members.

The children were encouraged to keep their data (photographs, drawings and participant grids) after the focus groups to reflect on the study; they were also told they could keep the sketchbooks for personal use after the study. The children were also informed during the focus groups that if they wished, they could be sent a report of the study's findings.

## Analysis

Recordings of the focus group were transcribed and checked for accuracy by the lead author. The transcripts, along with the complete set of photographs and drawings, were entered into NVivo to organise and categorise the data. Steps were taken to ensure quality of analysis; the lead author discussed coding of the data with the co‐authors to ensure analysis was as transparent as possible.

The analytic framework for the study was concurrent inductive and deductive thematic analysis (Fereday and Muir‐Cochrane, 2006); themes generated from the data were both data‐driven and theory‐driven. The visual data were analysed following two alternative processes. The lead author coded the visual data solely on location and subject matter. They were then analysed into themes by the children during the focus group.

The transcripts of the focus groups were read by the lead author who then sorted the verbal data into raw codes. Once every transcript had been coded, these raw codes were identified for patterns relating to the research aim; raw codes that followed similar patterns were placed together and arranged into first‐order themes. The same process then took place with the first‐order themes to place them into second‐order themes, which were then placed into overall themes. The overall themes have been further condensed into two groups; physical affordances and social affordances.

## Findings

### Physical affordances

The children spoke frequently about the lack of outdoor settings they felt they could go to, specifically places that contained perceived appropriate equipment for play. This was a recurrent issue within all three focus groups, and evident in both the visual and the verbal data. The data also suggested that children perceived that the play equipment in most places was designed for younger children and ‘not meant for them’. For example, Emily spoke of not liking the park because it was not exciting to her:

No, some parks are not very nice, but…

FH Why aren't they very nice?

Well they're not very exciting. They don't have much in them and my friends don't like going there so I'm not like accompanied by anyone. So I don't enjoy it as much.

FH So when you say there's not much there, what is there?

Well, not much there in terms of the stuff I like, really. There's like smaller, sort of smaller children's park. It's like, got like baby swings, and like a mini climbing frame thing. But I prefer to like just play football or something.

**Figure 2:**
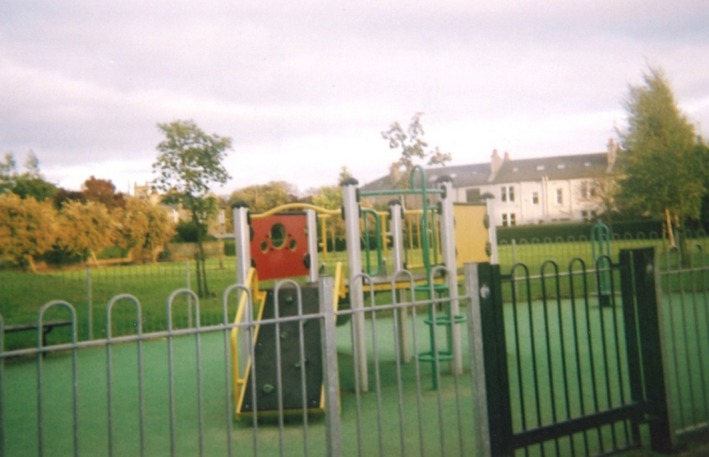
Robin's photograph showing equipment perceived as ‘too young’.

This issue was also emphasised by Robin who took a picture (Figure 2) of a local playground that was perceived as ‘too young’ for her to play on.

**Figure 3:**
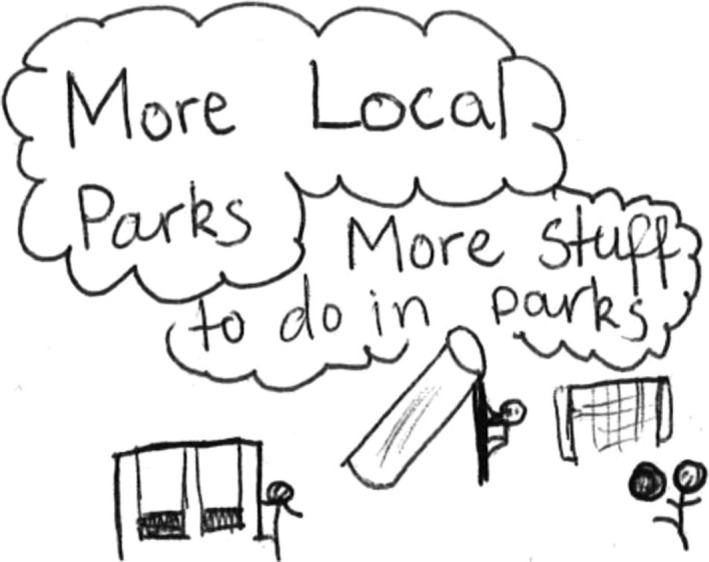
Elsa's drawing illustrating the need for more parks and equipment variety.

The girls, in particular, spoke frequently not only about the perceived lack of age appropriate equipment but also about the lack of equipment in general. The girls spoke of a desire for more equipment that they could use to play on. For example, Elsa drew a few pictures illustrating the need for more parks and more park equipment (Figure 3), while Jessica spoke of wanting bigger and more appropriate equipment:

FH Can you think of any equipment that you would want to play on, or that kids your age would like to play on?

Well like climbing frame, like bigger, big climbing frames, monkey bars, appropriate swings, that kinda thing.

**Figure 4:**
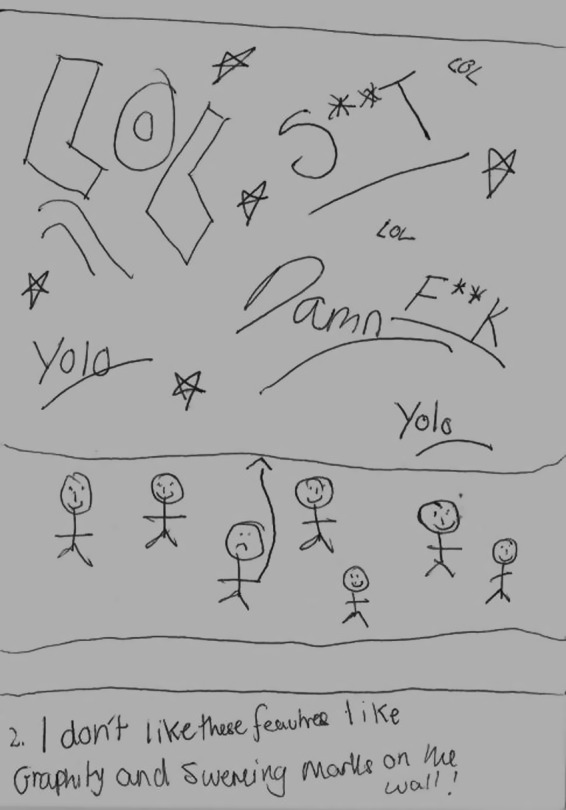
Paul's drawing illustrating illegal graffiti.

With regard to the aesthetic environment, many of the children depicted graffiti in a negative way, and wanted it to be removed (Figure 4).

**Figure 5:**
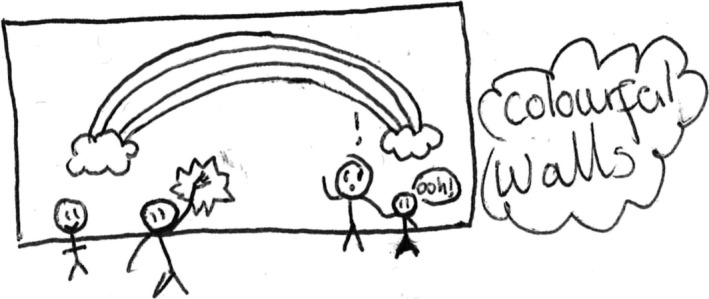
Ashley's drawing illustrating desire for a colourful environment.

**Figure 6:**
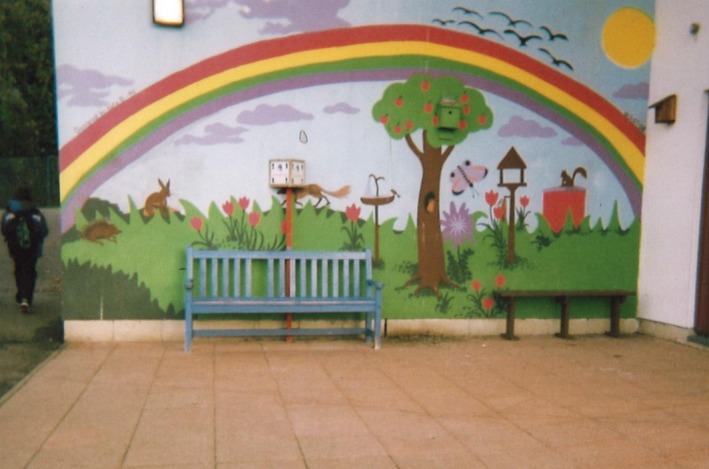
Rose's photograph illustrating colourful walls.

However, some of the children did suggest that their environment would be improved with the addition of colourful walls (Figures 5 and 6).

In the all‐boy focus group, the children discussed football as an activity they really enjoyed while they were outdoors. The children discussed numerous places where they played football; however, the most important environmental characteristic to the boys was that the location offered football‐related affordances, as mentioned by Ben:

We normally walk to basically there's, well there's a few parks near us but we normally just stick, go to ****.

FH Is that…? Why is that the one you go to the most?

Because that's the one with the best football pitch

Feeling safe was also a key factor when children talked about the places where they spent their time and why they did so. Rachel spoke about a ‘turning circle’ (a private space for cars to park or turn outside houses) outside her home that she liked to play in because she felt safe as she knew everybody in the surrounding area:

One of the places that I really like going is actually quite near my house. I have a huge turning circle – it's not supposed to be a turning circle, it's actually quite annoying people using it as a turning circle, cos we have to get out of the way and move all our stuff – but we really, me and my neighbours really enjoy playing there, and we normally use our bikes and scooters and we use our imagination and pretend games, and it's basically been something I've grown up with, so I know these people really well, and I know that nobody comes up here that scares me, because if they do then they're either in a car or they're just, they're just postmen or people giving out leaflets […] I feel safe there.

### Social affordances

During the focus groups, it appeared that friends were a key influence, in terms of which location the children would visit. For instance, if the child was with friends, they would require a different type of environment as the children sought alternative affordances depending on whether they were alone or with friends. Izzy discussed that she would visit a woodland area when she was with her friends because they could play hide‐and‐seek, but she would not go there by herself:

FH So, let's talk about this one, ‘Places I like going’.

Well this is a park, it's like a park but then it has like a really like good size wood next to it and I like to go there and play with my friends because it's fun and sometimes you don't know where you're going, which I also think is really fun. And you sort of just have to work your way around it like a maze, it's really fun to play with, with your friends, and I enjoy going there.

FH Ok, so if it was just a big open field, would we like it as much?

No, ‘cause it has like loads of trees and places that you can hide in and stuff […] I wouldn't go there by myself ‘cause it's quite scary by yourself.

FH Ok.

But I enjoy going there with my friends.

**Figure 7:**
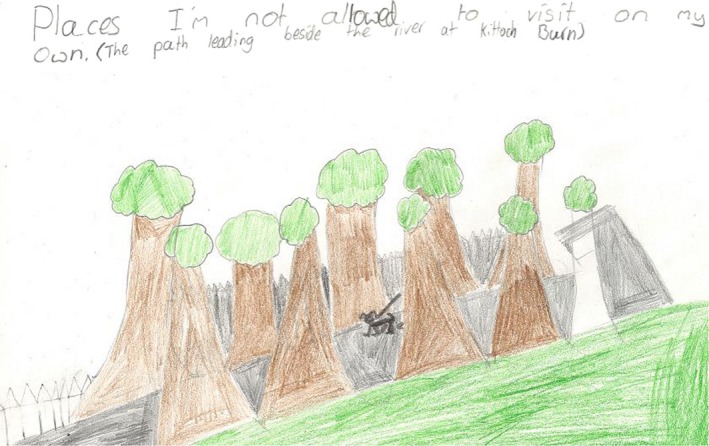
Drawing by Hector of a restricted location due to parental influences.

The children also spoke about and visually documented places where they were not allowed to go. These locations were frequently isolated paths or paths near dangerous locations, such as the following illustration (Figure 7) by Hector showing a location near a river.

The children also spoke of intimidating social environments that they avoided. ‘Hoodies’ were labelled as a type of teenager the children would avoid when deciding where to go in their environment. The following quote is from Luke, Sarah and Emily who define what a ‘hoodie’ is to them:

FH So hoodies are mentioned quite a lot (yeah) what about hoodies do you find intimidating?

Luke When they have their hood up.

FH So when you can't see their face?

Luke Yeah.

Emily Yeah.

Sarah Well, I can usually see their face it's just they walk about with like hoodies and trackies and like sort of junkies or something.

Luke Jakeys just like, look like they're gonna like come after you like to give them stuff. So some of them are quite intimidating.

FH So if you were know that they were gonna be in a certain park or in a certain place would you purposely avoid that place?

Luke Yes.

Ben and Luke also referred to teenagers ‘hanging out’ and considered this to be more intimidating than teenagers who were participating in a recognised activity such as football:

Ben You get the ones at the Peel who are in, colourful like football strips and they're just running around happy

Luke And you get the ones like what I was talking about at the parks, just in grey hoodies, and you can't see their face and then they've got jogging bottoms on and you just, it's like they just don't want you to see that they're hanging around there, but they kind of do because they want people to see that they're cool.

## Physical affordances

Affordances that offered play opportunities appeared to be a key determinant of where children spent their time in their environment. However, many of the children spoke of their local playgrounds having unsuitable equipment, which was perceived to be for much younger children. The study found that the children frequently felt they had nowhere to go — playgrounds were perceived as ‘too young’ for them which resulted in the children feeling the equipment was ‘not very exciting’. An outcome of this was children choosing to play in alternative areas such as a turning circle for cars. In a study by Veitch and others (2006), one parent noted that ‘we want to go to parks that are interesting. The closest park, we can walk to, but it does not interest my kids. It is a big park but the play equipment is too small and it only caters for younger children, seven‐ to eight‐year‐olds are not challenged there’ (p. 389). The problem of ‘boring’ playground equipment is one that has been around for many years. In 1999, Cunningham and Jones asked 26 children aged 10–13 years old to write short essays on the importance of play. The children did not mention (or rarely mentioned) playground equipment, when questioned; the children responded ‘they did indeed appreciate good equipment but a lot of it was boring’ (Cunningham and Jones, 1999, p. 13). Nearly 20 years later, children are reporting very similar findings, suggesting little has been done to rectify this problem.

One study has taken this line of inquiry further to establish whether children purposefully design more risky, less standardised play areas. Jongeneel and others (2015) conducted a study that explored whether children, when creating their own playground, opted for a less uniform set‐up. In Jongeneel's study, children elected for playground features that were not uniform. Furthermore, the children chose to create a playground that matched their action capabilities. In the current study, children felt the playgrounds available to them were too easy, and would prefer more ‘suitable’ equipment for their age, echoing the findings of Jongeneel and colleagues. The children discussed using a turning circle for cars as an area for play by using their ‘imagination’. It could be postulated that by doing this, the children were designing their own playground, and thus creating opportunities that matched their (perceived) action capabilities.

The finding of this study strongly suggests that children desire less safe, more risky playground equipment. Although research has found similar findings spanning over 20 years, it would appear little has been done to design playgrounds that challenge children in a way they would consider appropriate. From the findings of this study, and previous supporting evidence, we would suggest that policy‐makers reassess the design of playgrounds. It is important that when designing playgrounds for children, policy‐makers recognise the different action capabilities of all age groups, ensuring that the age group of 10–12 years are catered for.

## Social affordances

Other studies that were qualitative in nature have found similar results to the present study, for example, determinants such as parental restriction (Eyre and others, 2014), safety (Loureiro and others, 2010) and social intimidation (Brockman and others, 2011; Veitch and others, 2007) were all found to influence children's perceived barriers to spending time outside. As with the current study, Veitch and others (2007) found that younger children are aware of ‘stranger danger’ and this is a perceived barrier to spending time outside. A novel finding within the current study is that strangers (specifically teenagers) were perceived as less intimidating if they were taking part in a ‘recognised’ activity such as football. Teenagers who were perceived as just ‘hanging out’ were considered to be more intimidating and therefore, more of a barrier to the children using the space. Although perceived social intimidation as a barrier has been found frequently in research (Brockman and others, 2011; Veitch and others, 2007), the understanding that children are affected by whether the stranger in question is participating in a ‘known activity’ is particularly novel and highlights the importance of context when discussing social intimidation.

The current study has found evidence to suggest a relationship between friends and PA behaviours, a finding frequently supported by previous research (Edwards and others, 2015; Jago and others, 2009; Macdonald‐Wallis and others, 2012). The children spoke of spending more time outside if they were with their friends, and that if they could not find their friends, or their friends were busy, they would not go outside. The study did not present any original findings regarding the relationship between PA and friends. However, in relation to affordances, the children noted that they required different types of play affordances dependent on whether they were with their friends. When designing interventions to increase outdoor play, policy‐makers might think to provide different areas that cater to children who are with friends, and children who are by themselves.

One of the interesting findings emerging from the study was the children's views of graffiti. Consistent with previous literature, the children noted that they avoided locations they perceived to be unclean, such as places with litter, dog foul, and graffiti. There have been studies to suggest there is a negative association between children's environmental safety perceptions and incivilities (Rossen and others, 2011) as well as PA levels and neighbourhood incivilities (Ding and others, 2011). However, the current study found evidence to suggest graffiti may not always be considered negative. There appeared to be perceptive discrepancies between negative graffiti (commonly consisting of slang and swear words) and neighbourhood‐enhancing graffiti that was perceived as ‘colourful’. In the literature, graffiti is often related to the broken window theory, in that where there is graffiti, there is crime and violence (Wilson and Kelling, 1982). However, graffiti comes in many forms, ‘street art’ is often used to replace the term graffiti, when the work is perceived to be artistic and aesthetically appealing (Hughes, 2009). There are now numerous projects where graffiti is being used to help at‐risk youth and to improve community appearances, for instance, The Graffiti Transformation Project in America, where youths are encouraged to paint murals and change graffiti into works of art (http://www.stchrishouse.org). There is also the Graffiti Arts Project in America, where a police department has partnered with a local arts organisation. The aim of the intervention is teaching graffiti as an art form in an effort to reduce graffiti‐related crimes in the City, and to provide at‐risk youth positive alternatives to gangs and illegal activities (http://www.muralmusicarts.org).

The current study found evidence to suggest children would enjoy spending time in a ‘colourful’ environment, with the visual data showing colourful wall art. Moreover, the current study also found many teenagers to be intimidating when they ‘hung around’. As youth are often considered responsible for most graffiti (Klingman and others, 2000; Ten Eyck, 2016), there could be a case for allowing teenagers to artistically express themselves through legal wall graffiti that was socially acceptable in designated areas. This could effectively ‘kill two birds with one stone’; potentially lessening the number of teenagers ‘hanging around’, and create a colourful environment for the children.

## Conclusion

The study highlights the importance of taking a methodological approach, whereby children are fully involved in the study. Previous work in the field has steered away from including children in the data collection process. For instance, Teedon and others (2014) chose not to include children in their study for fear they ‘might find the connections between the environment and health too abstract and thus difficult to deal with’ (p. 51). This study provides a strong case that a combination of visual and verbal methods is a valid approach when exploring children's perceptions of the public outdoor environment.

As far as we are aware, no other studies in childhood literature have employed participant analysis for visual data. The most common criticism of visual data is that it is susceptible to researcher bias as pictures and drawings are difficult to interpret correctly. The lead author of this paper designed the participant analysis grid as a way of circumventing bias. The children were freely able to place their pictures in whichever box they felt represented their data and the children were also given the opportunity to create their own box if the pre‐labelled ones were not representative.

The idea that the children recognised which areas needed improvement and how they could be improved gives justification for policy‐makers and practitioners to consult with children of this age on how to make a more appealing environment for children. The participatory approach helped to further challenge traditional knowledge hierarchies providing justification that children of this age are able to comprehend the research process and add valuable contribution to the study at various stages.

One potential issue affecting the use of visual data is when there is an absence of conceptual or theoretical framework as it cannot be critically analysed without contextual knowledge (Harrison, 2002). In our study, this potential limitation was circumvented by acknowledging our use of Gibson's theory of affordances. Furthermore, the visual data were analysed by the children, limiting researcher bias.

Seasonality may have affected the findings of the study; the first two focus groups (September–October) produced high‐quality images, whereas images from the last focus group (late November) were lower in quality due to limited lighting. Seasonality may also have played a part in how the children perceived the outdoor environment. The study was conducted during the winter months; should the study be replicated during the summer, the children's perceptions of certain affordances may change.

The findings of this study support the enhancement of children's play spaces. Children between the ages of 10 and 12 years of age may lack suitable play affordances. Policy‐makers could provide more play opportunities by providing more risky, or challenging playground equipment. More research may need to be conducted to clarify specifically the precise equipment wanted by children. Creating places for specific age groups may help to encourage children to spend time being physically active within their neighbourhoods without being concerned or intimated by older children/young people. This could be hard to do as it would be difficult to exclude older teenagers from public spaces; however, more research may provide insight into ways of feasibly providing such spaces. The children spoke of needing alternative affordances if they were with friends. To encourage more PA out of school areas, there may need to be more affordances that encourage multi‐person play. For example, one child noted that the presence of bushes and trees allowed for hide‘n'seek. Many current greenspaces are wide and open; providing more affordances within these greenspaces may encourage more free play with friends.

The current study was an exploratory pilot study. The study provided the authors with valuable information regarding the feasibility of methods, participant analysis and how to design the focus group discussion. The valuable data generated in this study justify further work with children from different contexts (such as area deprivation) to better understand how children from different subgroups perceive their environment and how perceived affordances influence time spent outside.
